# The *AtCathB3* gene, encoding a cathepsin B-like protease, is expressed during germination of *Arabidopsis thaliana* and transcriptionally repressed by the basic leucine zipper protein GBF1

**DOI:** 10.1093/jxb/eru055

**Published:** 2014-03-05

**Authors:** Raquel Iglesias-Fernández, Dorothee Wozny, Maite Iriondo-de Hond, Luis Oñate-Sánchez, Pilar Carbonero, Cristina Barrero-Sicilia

**Affiliations:** Centro de Biotecnología y Genómica de Plantas (UPM-INIA), and E.T.S.I. Agrónomos, Campus de Montegancedo, Universidad Politécnica de Madrid, Pozuelo de Alarcón, 28223-Madrid, Spain

**Keywords:** *Arabidopsis thaliana*, *AtCathB3*, bZIP, cathepsin B-like enzymes, GBF1, phylogenetic shadowing, reserve mobilization, seed germination, transcriptional regulation.

## Abstract

Protein hydrolysis plays an important role during seed germination and post-germination seedling establishment. In *Arabidopsis thaliana*, cathepsin B-like proteases are encoded by a gene family of three members, but only the *AtCathB3* gene is highly induced upon seed germination and at the early post-germination stage. Seeds of a homozygous T-DNA insertion mutant in the *AtCathB3* gene have, besides a reduced cathepsin B activity, a slower germination than the wild type. To explore the transcriptional regulation of this gene, we used a combined phylogenetic shadowing approach together with a yeast one-hybrid screening of an arrayed library of approximately 1200 transcription factor open reading frames from *Arabidopsis thaliana*. We identified a conserved *CathB3-element* in the promoters of orthologous *CathB3* genes within the Brassicaceae species analysed, and, as its DNA-interacting protein, the G-Box Binding Factor1 (GBF1). Transient overexpression of GBF1 together with a *PAtCathB3::uidA* (β-glucuronidase) construct in tobacco plants revealed a negative effect of GBF1 on expression driven by the *AtCathB3* promoter. In stable *P35S::GBF1* lines, not only was the expression of the *AtCathB3* gene drastically reduced, but a significant slower germination was also observed. In the homozygous knockout mutant for the *GBF1* gene, the opposite effect was found. These data indicate that GBF1 is a transcriptional repressor of the *AtCathB3* gene and affects the germination kinetics of *Arabidopsis thaliana* seeds. As *AtCathB3* is also expressed during post-germination in the cotyledons, a role for the AtCathB3-like protease in reserve mobilization is also inferred.

## Summary

The cathepsin B-like protease-encoding gene *AtCathB3* positively influences germination in *Arabidopsis* seeds, and GBF1 has been identified as its transcriptional repressor that negatively affects the process.

## Introduction

Seed germination begins with water uptake and is completed when the radicle protrudes through the layers surrounding the embryo (germination *sensu stricto*). The protrusion also marks the onset of post-germination events, where seedling growth is mainly supported by the hydrolysis of nutritional reserves until it becomes a photosynthetic competent entity. Seed germination and storage reserve mobilization are two distinct, highly controlled processes, independently regulated and influenced by genetic and environmental factors (Bewley, 1997; Pritchard *et al.*, 2002; Vicente-Carbajosa and Carbonero, 2005; Finch-Savage and Leubner-Metzger, 2006; Nonogaki *et al.*, 2007; Holdsworth *et al.*, 2008; Piskurewicz *et al.*, 2009; Iglesias-Fernández *et al.*, 2011). Brassicaceae mature seeds, such as those of *Arabidopsis thaliana*, mainly accumulate reserves as lipids and seed storage proteins (SSPs). SSPs are deposited in protein storage vacuoles making up the protein bodies of the cotyledons and the embryonic axis (Jiang *et al.*, 2001; Hills, 2004; Tan-Wilson and Wilson, 2012). During imbibition of these seeds, protein degradation begins at the radicle tip and continues at the pre-vascular strands and subepidermal cell layers where cell expansion and differentiation are being initiated. In the post-germination period, the hydrolysis of SSPs occurs mainly in the cotyledons when proteins of the embryonic axis are nearly exhausted (Müntz, 2007).

Cathepsin B-like cysteine proteases (EC 3.4.22.1; CysProt) belong to the Papain-like class of CysProt (MEROPS Peptidase Database; Rawlings *et al.*, 2012) and are endopeptidases preferentially associated with SSP degradation and mobilization during seed germination and post-germination (García-Lorenzo *et al.*, 2006; Tan-Wilson and Wilson, 2012). These peptidases are synthesized as inactive precursors (zymogens) that are converted into active enzymes by the proteolysis of an N-end peptide (Khan and James, 1998). Upon seed imbibition, SSP mobilization occurs in part by zymogen activation, but during post-germination the major SSP hydrolysis occurs due to the action of *de novo*-synthesized proteases in the cotyledons. However, transcriptomic analyses of *Arabidopsis* seeds have shown that during the early stages of germination, genes involved in protein synthesis and degradation are over-represented, reflecting the importance of protein turnover throughout this process (Nakabayashi *et al.*, 2005).

Phytohormones, mainly gibberellins (GA) and abscisic acid (ABA) orchestrate the progression of seed germination (Nambara *et al.*, 2010). The endogenous ABA content in dry seeds rapidly declines upon imbibition and, by contrast, GA synthesis increases rapidly during the early and the late phases of germination (Holdsworth *et al.*, 2008; Weitbrecht *et al.*, 2011). In *Arabidopsis*, germination *sensu stricto* is inhibited by ABA, but this hormone seems not to affect lipid mobilization (Pritchard *et al.*, 2002). In cereal aleurone layers, the synthesis of hydrolases, such as CysProt, takes place mainly during post-germination and is activated in response to GA diffused from the embryo. These enzymes are secreted into the endosperm where they mobilize the bulk of the stored reserves (Sun and Gubler, 2004; Eastmond and Jones, 2005). Functional analyses of the promoters of hydrolase genes induced by GA in the barley aleurone (Cejudo *et al.*, 1992; Gubler *et al.*, 1999) have identified a conserved tripartite *cis*-acting GA-responsive complex. This complex comprises the GARE motif (GA-responsive element, 5′-TAACAAA-3′), the pyrimidine box (5′-CCTTTT-3′), and the 5′-TATCCAC-3′ box, which are recognized by transcription factors (TFs) belonging to the R2R3MYB, DOF, and R1MYB (SHAQKYF) families, respectively (Gubler *et al.*, 1995; Mena *et al.*, 2002; Isabel-Lamoneda *et al.*, 2003; Rubio-Somoza *et al.*, 2006; Moreno-Risueño *et al.*, 2007). However, the GA-responsive complex has not been described in promoters of hydrolase genes induced upon germination of dicotyledoneous seeds (Holdsworth *et al.*, 2008; Weitbrecht *et al.*, 2011; Iglesias-Fernández *et al.*, 2013).

In this paper, we explore the physiological significance and transcriptional regulation of *AtCathB3*, a gene that encodes a cathepsin B-like protease, during *Arabidopsis thaliana* seed germination and post-germination periods. *AtCathB3* transcripts are the most abundant *CathB-like* mRNAs throughout both periods. Homozygous germinating seeds for a knockout (KO) in this *AtCathB3* gene present a significantly slower germination besides having a decreased cathepsin B-like protease activity compared with that of the wild type (Wt). The transcriptional regulation of this gene has been further explored and the G-box Binding Factor 1 (GBF1) has been identified as a transcriptional repressor of the *AtCathB3* gene. The regulatory function of GBF1 upon *AtCathB3* gene expression is validated by *trans*-activation assays in tobacco leaves, real-time quantitative PCR (RT-qPCR) analyses, and mRNA fluorescence *in situ* hybridization (FISH) experiments, and also by the germination kinetics of both overexpressor (oex) and T-DNA KO insertion lines in the *GBF1* gene. The oex-*GBF1* lines not only have a significant reduction of the *AtCathB3* gene expression levels upon seed germination, but these seeds also germinate slower than the Wt seeds. The opposite behaviour is observed in the KO *gbf1* mutant seeds.

## Materials and methods

### Plant material and germination assays

Plant growth conditions were as follows: ethanol sterilization of *Arabidopsis* seeds was done for 20min before germination in Petri dishes containing 0.5× MS medium including vitamins (Duchefa-Biochemie, Haarlem, The Netherlands) and solidified with 0.8% agar. Immediately after plating, seeds were stratified for 3 d at 4 °C and then incubated at 21 °C under long-day conditions (16h/8h light/dark; light intensity: 155 μmol photons m^–2^ s^–1^) for 2 weeks and then transferred to pots in the greenhouse. Mature seeds for Wt and homozygous for T-DNA insertion and oex mutants were stored at 21 °C and 30% relative humidity, until used for germination assays.

Three replicates of 50 after-ripening seeds were germinated in 90mm Petri dishes on two layers of filter paper (Whatman No. 1) moistened with 3ml of sterile water. Germination was done at 21 °C in growth chambers under long-day conditions as described above, and seeds were always sown in the morning. Seeds were neither surface sterilized nor stratified at 4 °C in order to avoid influencing their dormancy status; nevertheless, fungal infections were not detected by light microscopy during the studied period. Seeds were scored as germinated when the radicle emergence through the seed coat was visible under a magnifying lens. Germination tests were performed in three biological samples using three technical replicates. Statistical analyses were done using the GERMINATOR package program (http://www.pph.wur.nl/UK/seedlab/resources/germinator; Joosen *et al.*, 2010).

### Generation of transgenic lines

All material used in this study was derived from *Arabidopsis thaliana* ecotype Col-0. The T-DNA insertion mutant in *AtCathB3* exon 8 (SALK_019630) was obtained from the Nottingham Arabidopsis Stock Centre, and homozygous plants were selected by PCR using a gene-specific primer and a primer derived from the left border of the T-DNA (Supplementary Table S1 available at *JXB* online; http://www.signal.salk.du/tdnaprimers.2.html). The homozygous T-DNA insertion line in *GBF1* exon 9 (SALK_144534) was kindly provided by Dr Zengraft from University of Tubingen, Germany (Smykowski *et al.*, 2010).

The *AtCathB3* promoter (–500bp) was amplified from genomic DNA by nested PCR using oligonucleotide pairs containing *attB* sites (Supplementary Table S1 available at *JXB* online) for cloning into the pDONOR^®^207 vector by the Gateway^*®*^BP and then transferred by Gateway^*®*^ LR recombination (Invitrogen, Carlsbad, CA, USA) into the destination vector pMDC163, containing the *uidA* reporter gene (Curtis & Grossniklaus, 2003). In the T1 generation, plants carrying a pMDC plasmid were identified on the basis of hygromycin resistance. The next generation was tested for single-locus insertion of the transgene based on a 3:1 segregation in MS medium containing hygromycin. Homozygous lines were selected in the T3 generation. The same strategy was used to obtain the oex lines. *P35S::GBF1* was cloned into pMDC43, using the plasmid pDEST22^*®*^ containing the GBF1 open reading frame (ORF), as the entry clone. All constructs were introduced into *Agrobacterium tumefaciens* strain C58C1 GV3101 by electroporation and these were used to transform *Arabidopsis thaliana* (Col-0) by the floral dip method (Clough and Bent, 1998).

### Histochemical β-glucuronidase (GUS) assays

Qualitative GUS staining assays were performed using the protocol described by Jefferson *et al.* (1987). Samples were cleared using Hoyer’s light medium as described by Stangeland and Salehian (2002). GUS histochemical staining was visualized under a light stereomicroscope (Leica, Wetzlar, Germany). A representative transgenic line out of 10–12 independent lines was used.

### Protease activity assays

Three lots of non-stratified seeds germinated for 12, 24, 36, 48 and 60h were ground under liquid nitrogen and then extracted with protein extraction buffer (340mM sodium acetate, 60mM acetic acid, 4mM EDTA, 8mM dithiothreitol, pH 6). The enzymatic activity of protein extracts was determined using a fluorogenic specific substrate for cathepsin B proteases, Z-Arg-Arg-7-amido-4-methylcoumarin (Z-RR-AMC), following the manufacturer’s instructions (Calbiochem, San Diego, CA, USA). Mixtures of 20 µg of protein extracts and substrate (20 µM) were incubated at 30 °C for 2h and the emitted fluorescence was measured at 20min intervals with a 365nm excitation wavelength and a 465 emission wavelength.

### Bioinformatic tools: dendrogram and phylogenetic shadowing

Sequences from the six different species within the Brassicaceae family used in this work were obtained from the Phytozome v8.0 Database (Goodstein *et al.*, 2012; http://www.phytozome.net), except for those of the *Arabis alpina* genome that were produced by the TRANSNET KBBE consortium (Professor P. Carbonero, Universidad Politécnica de Madrid, Madrid, Spain; personal communication). The major characteristics of the predicted cathepsin B-like proteins are listed in Supplementary Table S2 available at *JXB* online. The complete deduced amino acid sequences of the 14 *CathB-like* genes from the six Brassicaceae species were used to reconstruct a phylogenetic dendrogram using the neighbour-joining algorithm (Supplementary Fig. S1 available at *JXB* online).

The promoter sequences of the six Brassicaceae *CathB3* orthologous genes from *Arabidopsis thaliana*, *Arabidopsis lyrata*, *Capsella rubella*, *Arabis alpina*, *Brassica rapa* and *Eutrema salsugineum* are given in Supplementary Table S3 available at *JXB* online. The mVISTA Shuffle-LAGAN software (http://genome.lbl.gov/vista/index.shtml; Frazer *et al.*, 2004) was used to create pairwise alignments between promoter sequences, and the conserved regions were analysed with MUSCLE (http://www.drive5.com/muscle; Edgar, 2004) and T-Coffee programs (http://www.ebi.ac.uk/Tools/msa/tcoffee; Notredame *et al.*, 2000). Plant *cis*-acting core regulatory DNA elements were searched through the following data bases: MotifFinder (http://www.genome.jp/tools/motif), BIOBASE (http://www.biobase-international.com), and PlantCARE (Lescot *et al.*, 2002).

### Yeast one-hybrid (Y1H) assays

Y1H screenings were performed as described previously (Castrillo *et al.*, 2011). The *CathB3-element* was amplified by PCR using specific primers that contained *Xma*I and *Xba*I restriction sites (Supplementary Table S1 available at *JXB* online). The engineered restriction sites of the *CathB3-element* were used to clone it into the *Xma*I and *Xba*I sites of the pTUY1H plasmid upstream of a *HIS3* reporter gene. The plasmid was introduced into *Saccharomyces cerevisiae* Y187α (MAT-α) cells. As a prey, a yeast-normalized library of *Arabidopsis thaliana* TFs containing a collection of approximately 1200 TF ORFs fused to GAL4AD arrayed in a 96-well format into *S. cerevisiae* YM4271 (MAT-a) was screened. Diploid cells, grown in selection medium lacking leucine (L) and tryptophan (W), were spotted onto plates with the screening medium devoid of L, W and histidine (H) to which increasing concentrations of 3-amino-1,2,4-triazole (3-AT; Sigma, St Louise, MO, USA) were added. Positive colonies were visible after 2–5 d of incubation at 28 °C.

### Transient expression assays

For transient expression in tobacco, *Nicotiana benthamiana* leaves were infiltrated with *Agrobacterium tumefaciens* (strain C58C1 GV3101) that had previously been transformed with the corresponding reporter (*PAtCathB3::uidA*) and effector (*P35S::GBF1*) constructs, or with an empty *P35S* vector as negative control. *Agrobacterium tumefaciens* containing *pMDC32-P35S::LUC* [luciferase (LUC); Gateway Technology, Invitrogen] for normalization and *pBIN61-P35S::P19* to avoid silencing (Voinnet *et al.*, 2003) were added. Relative GUS/LUC activities were determined as described by Jefferson *et al.* (1987) using a Genios Pro 96/384 multifunction microreader (TECAN^®^; Tecan Group, Männedorf, Switzerland). A minimum of three independent transformation experiments for each construct was done.

### RT-qPCR assays

Total RNA was purified, as described previously (Oñate-Sanchez and Vicente-Carbajosa, 2008), from different organs of *Arabidopsis* 5-week old plants: rosette leaves, roots, stems, and flowers. Siliques at different stages of development and seeds at different germination time points (up to 48h) were also analysed. cDNA was synthesized from 1 µg of total RNA using a First Strand cDNA Synthesis kit for RT-PCR (Roche Applied Science, Mannheim, Germany) following the manufacturer’s instructions. Samples were stored at –20 °C until used.

The specific primers used in the RT-qPCR assays are shown in Supplementary Table S4 available at *JXB* online, and the ubiquitin gene *UBC21* (*At5g25760*) was used to normalize the data, as it has been demonstrated to be constant throughout the periods studied, as was gene *ACT8* (*At1g49240*; Graeber *et al.*, 2011; Supplementary Fig. S2 available at *JXB* online). Amplification was performed in an Eco^®^ Real-Time PCR System (Illumina, San Diego, CA, USA). For each 10 µl reaction, 2 µl of the sample’s cDNA was mixed with 5 µl of FastStart SYBR Green Master (Roche Applied Science) and 0.25 µl of each primer (final concentration 500nM), plus sterile water up to the final volume. Samples were subjected to thermal-cycling conditions of 95 °C for 10min, and 40 cycles of 10 s at 95 °C and 30 s at 60 °C for annealing and extension. The melting curve was designed to increase from 55 to 95 °C, and melting temperatures for each amplicon and primer efficiencies were estimated using a calibration dilution curve and slope calculation (Supplementary Table S4 available at *JXB* online). Three different biological samples for each time point were considered, and expression levels were determined as the number of cycles needed for the amplification to reach a cycle threshold fixed in the exponential phase of the PCR (Pfaffl, 2001).

### FISH experiments

The protocol followed was as described by Testillano and Risueño (2009). Digoxygenin-labelled RNA (200–300bp) derived from the 3′ non-coding regions of the *Arabidopsis* genes analysed was used as a probe (Supplementary Table S1 available at *JXB* online). After incubation with an alkaline phosphatase-conjugated anti-digoxigenin antibody, the sections were incubated with a secondary antibody, anti-mouse IgG conjugated to Alexa Fluor 488 (Invitrogen). After washing in PBS, the nuclei were stained with 4′,6-diamidino-2-phenylindole (DAPI), washed in sterile water, and mounted and examined in a confocal microscope (Leica SP8; Leica).

## Results

### The *AtCathB3* gene is highly expressed in *Arabidopsis thaliana* seeds upon germination

**Fig. 1.**
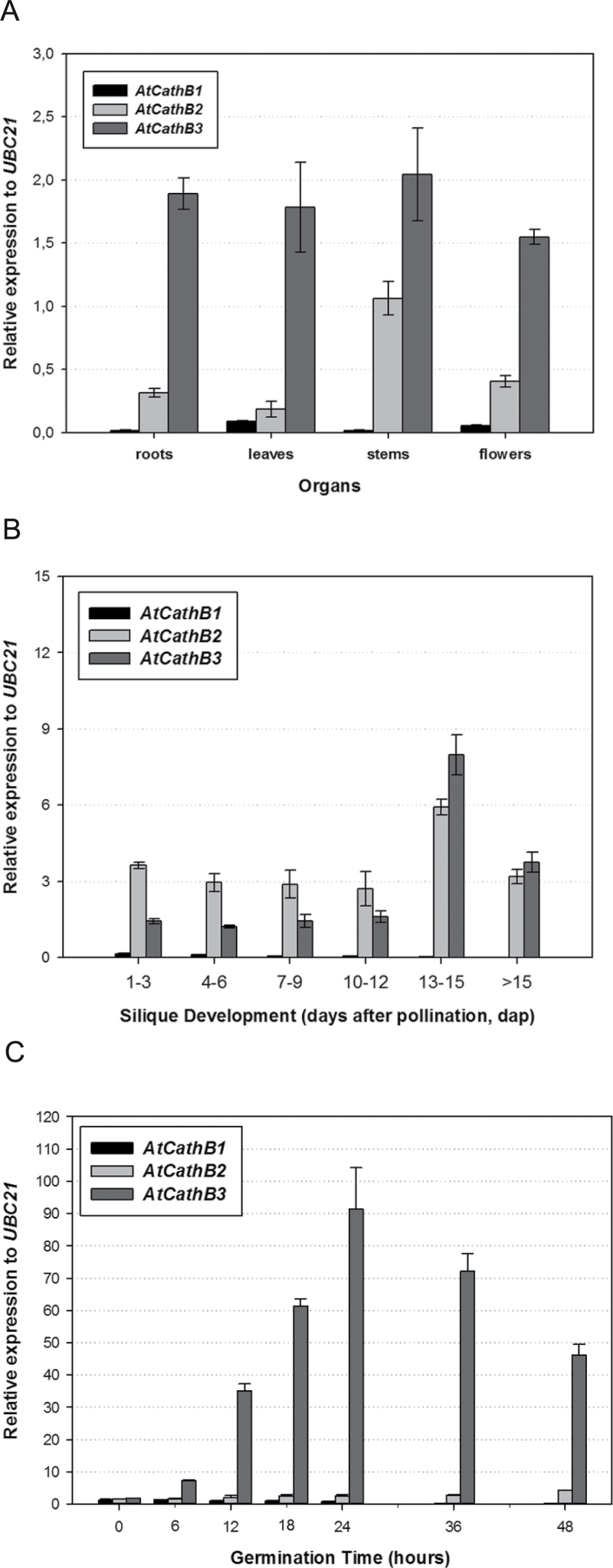
Expression analyses of the *AtCathB* genes by RT-qPCR. (A) Expression of *AtCathB1*, *AtCathB2*, and *AtCathB3* in different organs: roots, rosette leaves, stems, and flowers. Plant material was harvested from 5-week old plants. (B) Expression of *AtCathB1*, *AtCathB2*, and *AtCathB3* throughout silique development: at 1–3, 4–6, 7–9, 10–12, 13–15, and >15 dap. (C) Expression of *AtCathB1*, *AtCathB2*, and *AtCathB3* during seed germination at 0 (dry seeds), 6, 12, 18, 24, 36 and 48h. Data were normalized to the *UBC21* gene and are shown as means±standard error (SE) of three independent experiments.

Three *CathB-like* genes, *AtCathB1*, *AtCathB2*, and *AtCathB3* (loci *At1g02300*, *At1g02305*, and *At4g01610*, respectively) have been annotated in the *Arabidopsis thaliana* genome (McLellan *et al.*, 2009). The expression profiles of these *AtCathB* genes have been analysed by RT-qPCR in different *Arabidopsis* organs from 5-week old plants: roots, rosette leaves, stems, and flowers. The data in Fig. 1A show that, whereas the *AtCathB2* and *AtCathB3* transcripts were detected in all the organs analysed, *AtCathB1* was only faintly expressed. Siliques at different stages of development (1 to >15 d after pollination, dap) were analysed, and again, while the transcripts of *AtCathB1* were almost undetectable, those of *AtCathB2* and *AtCathB3* appeared throughout fruit development, reaching a peak between 13 and 15 dap (Fig. 1B). The three *AtCathB* gene transcripts were detected at the same level (<2 relative to *UBC21*) in dry seeds (Fig. 1C), but *AtCathB3* gene expression was highly induced upon germination (>50 times compared with the dry stage), reaching a peak at 24h and still being expressed at 48h when germination was completed (100% radicle emergence).

**Fig. 2.**
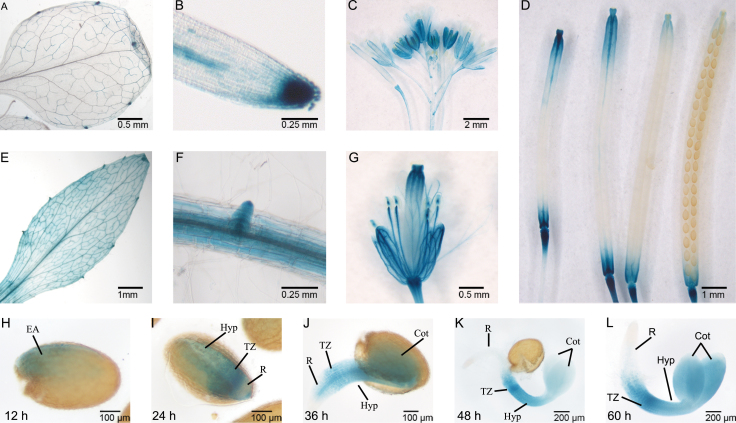
Histochemical localization of GUS expression in *Arabidopsis*-transformed plants with the *PAtCathB3::uidA* construct. (A) Young leaf; (B) radicle; (C) inflorescence; (D) siliques; (E) adult leaf; (F) secondary root; (G) flower; (H–L) germinating seeds: at 12 (H), 24 (I), 36 (J), 48 (K), and 60 (L) h. EA, embryonic axis; Hyp, hypocotyl; TZ, transition zone; R, root; Cot, cotyledon.

To analyse further the expression of the *AtCathB3* gene, stable transgenic lines of *Arabidopsis thaliana* (Col-0) with a construct of its promoter (–500bp) transcriptionally fused to the reporter *uidA* gene, which encodes GUS, were produced (*PAtCathB3::uidA*). GUS histochemical staining of these lines confirmed the ubiquitous expression of the *AtCathB3* gene (Fig. 2). In young and adult leaves, GUS expression was particularly intense at the hydathodes and at the vascular tissue (Fig. 2A, E). In roots, GUS activity was detected in the meristematic cells and at the radicle tip, in the incipient vascular elements, and at the initiation of the secondary roots (Figs. 2B, F). GUS was also expressed at the vasculature of the sepals and anthers, and at the stigma of flowers (Fig. 2C, G), and in fruits in the vascular bundles at the base and at the upper part of the silique (Fig. 2D). Upon germination, GUS activity in the *PAtCathB3::uidA* reporter lines was already detected before radicle protrusion, in the hypocotyl–radicle transition zone of the embryonic axis (12–24h), and later (36–60h) the signal was extended throughout the incipient vascular elements of the radicle and the hypocotyl, as well as to the cotyledons, where GUS expression was enhanced as the cotyledons expanded (Fig. 2H–L).

### Seed germination is affected in a homozygous KO mutant of the *AtCathB3* gene

**Fig. 3.**
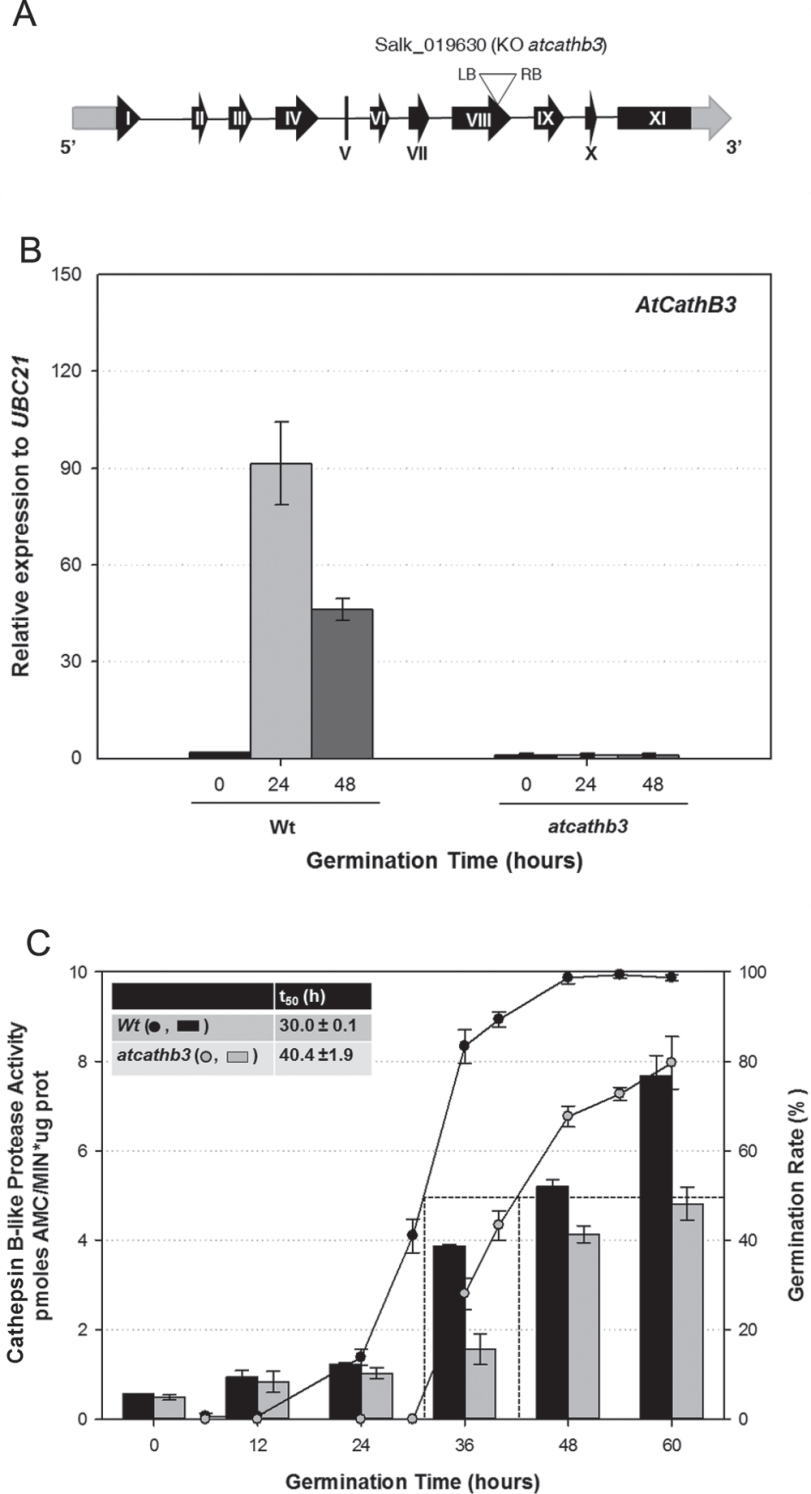
The mutant line for *AtCathB3* (KO *atcathb3*) is negatively affected in CathB protease activity and germinates slower than the Wt seeds. (A) Position of the T-DNA insertion, indicated with a triangle in exon VIII, in the *AtCathB3* mutant analysed (KO *atcathb3*). (B) Transcript analysis by RT-qPCR of the *AtCathB3* gene in Wt and KO *atcathb3* lines during seed germination at 0 (dry seeds), 24 and 48h. Data were normalized to the *UBC21* gene and are shown as means±SE of three independent experiments. (C) Cathepsin B protease activity (per µg of total protein) in Wt (filled bars) and KO *atcathb3* (shaded bars) seeds during germination at 0 (dry seeds), 12, 24, 36, 48 and 60h. Lines indicate the germination kinetics of Wt (filled circles) and KO *atcathb3* (open circles). The inset shows *t*_50_ data (time to obtain 50% germinated seeds). KO *atcathb3* seeds reached 100% germination at 72h. Data are means±SE of three independent experiments.

To address the question of whether the *AtCathB3* gene has a functional role during the germination process of *Arabidopsis* seeds, a homozygous T-DNA KO line (SALK_019630; *atcathb3*, T-DNA insertion in exon 8) for this gene was produced and the lack of *AtCathB3* transcripts confirmed by RT-qPCR analysis (Fig. 3A, B). Whereas the Wt seeds had completed their germination by 48h, only 80% of the mutants had germinated at 60h (Fig. 3C). Moreover, the *t*_50_ (time to reach 50% of germinated seeds) was significantly delayed in the mutant (40.4±1.9h versus 30.0±0.1h in the Wt; Fig. 3C. inset). Although very low cathepsin B activity was detected in dry seeds, both in the Wt and in the mutant, this enzymatic activity increased upon germination and post-germination (from 24 to 60h) in the Wt seeds. However, in the homozygous *atcathb3* seeds, this increase was approximately 50% of that shown in the Wt at 60h. The cathepsin B activity detected in the mutant could be due to the truncated CathB3 protein generated by the T-DNA insertion in exon 8, as the cysteine active site is found in exon 6 (Supplementary Table S2 available at *JXB* online). These data indicated that the *AtCathB3* gene expression affects the germination rate, as well as the protease activity during this process.

### Identification of an evolutionarily conserved *cis*-element in the *AtCathB3* gene promoter and of a GBF1 protein recognizing the G-box motif within it

The *Arabidopsis thaliana* CathB-like family was used to search for orthologous genes in other related Brassicaceae species such as *Arabidopsis lyrata*, *Capsella rubella*, *Arabis alpina*, *Eutrema salsugineum*, and *Brassica rapa* (Supplementary Table S2 available at *JXB* online). The corresponding deduced protein sequences were used to reconstruct an unrooted phylogenetic tree (Supplementary Fig. S1 available at *JXB* online). The 14 sequences compared were grouped into two major clusters of orthologous genes, supported by bootstrap values >70%. The phylogenetic tree showed that *AtCathB1/AtCathB2* and *AlCathB1/AlCathB2* were paralogous, probably originating from a recent duplication event occurring after the diversification of the *CathB-like* genes into two major clusters of orthologous genes. For *AtCathB3*, only one orthologous gene from each species was found. All the CathB-like proteases annotated contained a peptidase C1A pro-peptide site and a peptidase C1A papain C-terminal domain (Supplementary Table S2 available at *JXB* online), predicted by the InterProScan program (Quevillon *et al.*, 2005).

**Fig. 4.**
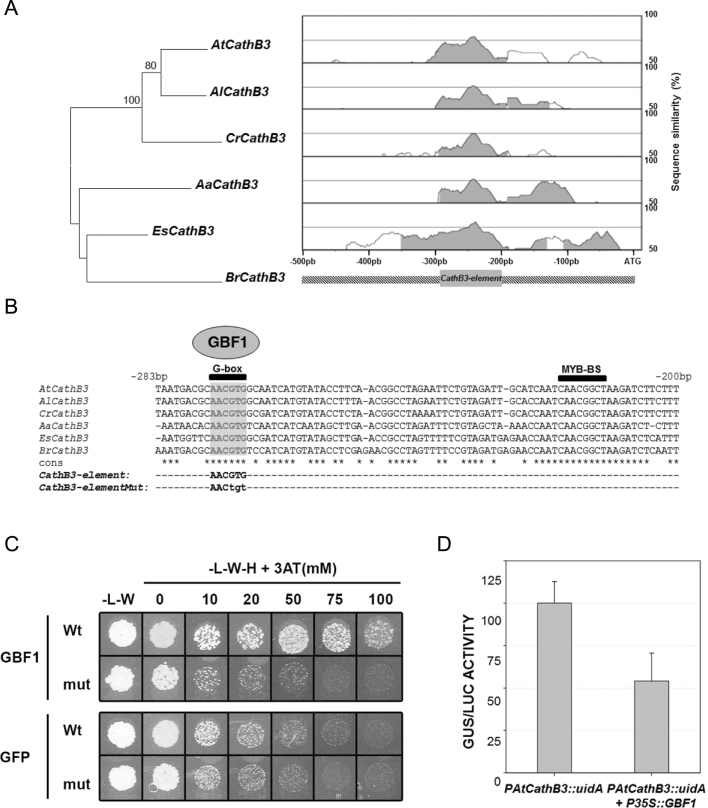
Identification of conserved *cis*-elements in orthologus CathB3 promoters in the Brassicaceae species: *Arabidopsis thaliana* (At), *Arabidopsis lyrata* (Al), *Capsella rubella* (Cr), *Arabis alpina* (Aa), *Eutrema salsugineum* (Es), and *Brassica rapa* (Br). The GBF1 transcription factor interacted with the CathB3-element in yeast one-hybrid screenings (Y1H) and negatively regulated the expression of the *PCathB3::uidA* construct in tobacco transient assays. (A) Pairwise alignment of CathB3 promoter sequences from different Brassicaceae species using mVISTA. For the graphical output, a sliding window of 100bp was used and base pair identity ranged from 50 to 100%. Shaded areas show conserved blocks. (B) Sequence alignment of the CathB-element (–283 to –200bp). A G-box and MYB-binding site (MYB-BS) core motives are indicated. The G-box core is shaded and a mutated version of the G-box (CathB3-element Mut) is indicated. (C) GBF1 specifically binds to the G-box sequence of the CathB3-element in a Y1H screening. Growth of diploid cells after mating of two yeast strains containing the *CathB3-element–pTUY1H* construct and *pDEST22-AD:GBF1* or *pDEST22-AD:GFP* (negative control) on non-selective (–L–W) or selective medium (–L, –W, –H) with increasing concentrations of 3-AT. (D) Tobacco leaves co-infiltrated with *Agrobacterium tumefaciens* containing *PCathB3::uidA* as reporter construct and *P35S::GBF1* as effector construct. GUS activity was relative to LUC activity, which was used as internal control. Data are means±SE of six biological replicates.

The promoter regions of the *CathB3* orthologous genes in the Brassicaceae species studied were searched *in silico* for conserved sequences through a phylogenomic approach (Vavouri and Elgar, 2005). The promoter regions compared spanned the intergenic distance between the ATG translation initiation codon of *AtCathB3* (*At4g01610*) and its preceding *GRAS* gene (*At4g01600*; Supplementary Table S2 available at *JXB* online). The pairwise alignment among the six promoters analysed using the mVISTA, MUSCLE, and T-Coffee programs (Notredame *et al.*, 2000; Edgar, 2004; Frazer *et al.*, 2004) showed a highly conserved block, with >70% identity, spanning from –283 to –200bp (Fig. 4A, ‘*CathB3-element*’). This *cis*-regulatory element contained two highly conserved core sequences (Fig. 4B): a G-box [binding site for basic leucine zipper (bZIP) proteins; Foster *et al.*, 1994] and a MYB-binding site (Prouse and Campbell, 2012).

To identify putative TFs able to interact with the *CathB3-element*, a library of approximately 1200 *Arabidopsis thaliana* TF ORFs fused to the GAL4 activation domain and arrayed in a 96-well format (RR library; REGIA+REGULATORS library) was used to perform a mating-based Y1H screening. The *CathB3-element* was cloned in the episomal plasmid (pTUY1H) to be used as a bait for the screening of this RR library (Castrillo *et al.*, 2011).

Following this procedure, the bZIP GBF1 (locus *At4g36730*) was identified. Diploid yeasts containing the plasmids *CathB3-element-pTUY1H* and *GBF1-pDEST22* were able to grow in an auxotrophic medium lacking histidine even in the presence of 100mM 3-AT, a competitive inhibitor of the product of the *HIS3* gene, while growth of the negative control (*CathB3-element–pTUY1H*+*GFP–pDEST22*) was inhibited at the lowest concentration of the inhibitor used (10mM 3-AT; Fig. 4C). To determine whether the binding of GBF1 is through the G-box element (*CathB3-element:* 5′-AACGTG-3′), a mutation was produced within this G-box (*CathB3-element* mut: 5′-AACtgt-3′; Schindler *et al.*, 1992*b*). The growth of diploid yeasts containing plasmids *GBF1–pDEST22* and *CathB3-element mut–pTUY1H* was inhibited at 3-AT concentrations similar to those inhibiting the green fluorescent protein (GFP) negative control (10mM and higher; Fig. 4C). Thus, the G-box-binding site contained in the *CathB3-element* was specifically recognized by the GBF1 TF.

The interaction between GBF1 and the *CathB3-element* was investigated *in planta* (Fig. 4D), using a tobacco transient expression system where *PAtCathB3::uidA* (GUS) was the reporter construct and a plasmid where GBF1 was under the control of the cauliflower mosaic virus (CaMV) 35S promoter, *P35S::GBF1*, acted as the effector. GUS activity (of at least six independently transformed plants) was measured in *Nicotiana benthamiana* leaves co-infiltrated with *Agrobacterium tumefaciens* containing the *PAtCathB3::uidA* reporter construct and *Agrobacterium tumefaciens* containing either a *P35S::GBF1* construct or the corresponding empty vector as negative control. A construct containing the CaMV 35S promoter fused to the LUC-encoding ORF was used as an internal control for the transformation experiments. The GUS activity in plants co-transformed with the reporter construct and the empty negative-control vector defined the basal GUS activity (100%). Co-transformation of the reporter construct together with the *P35S::GBF1* effector reduced the GUS activity, driven by the *AtCathB3* promoter, by about 50% (Fig. 4D).

### GBF1 is a transcriptional repressor of the *AtCathB3* gene and negatively affects *Arabidopsis thaliana* seed germination

**Fig. 5.**
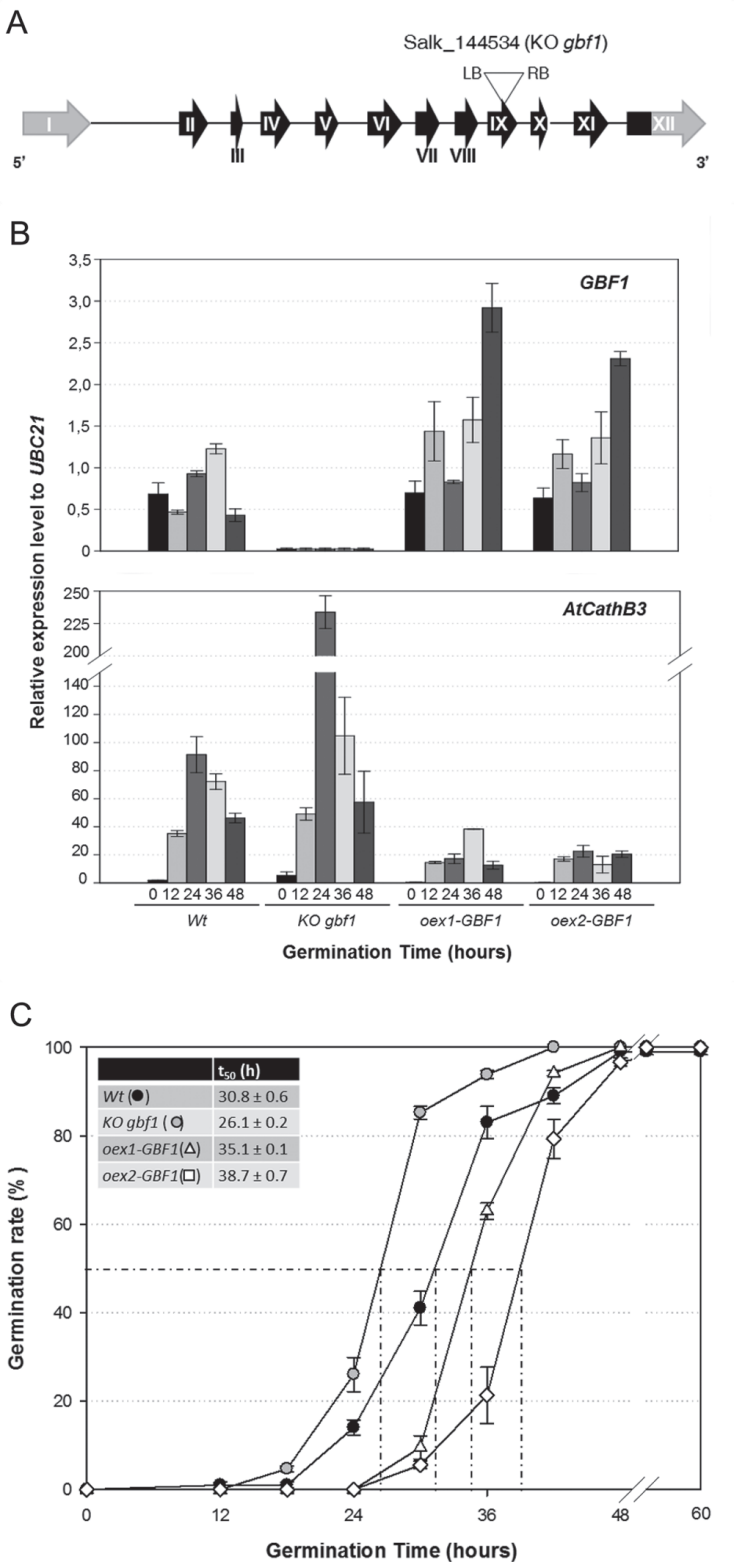
*AtCathB3* gene expression and germination kinetics in two over-expressor lines (*oex1-GBF1*, *oex2-GBF1*) and a KO line for *GBF1* (*gbf1*). (A) Position of the T-DNA insertion is indicated with a triangle in exon IX of *GBF1* (*gbf1* mutant). (B) Transcript analysis by RT-qPCR of the *GBF1* and *AtCathB3* genes during germination of Wt, KO *gbf1*, *oex1-GBF1*, and *oex2-GBF1* seeds at 0 (dry seeds), 12, 24, 36, and 48h. Data were normalized to the *UBC21* gene and are shown as means±SE of three independent experiments. (C) Germination kinetics of seeds from: Wt (filled circles), KO *gbf1* (shaded circles), *oex1-GBF1* (triangles), and *oex2-GBF1* (squares). Data are means±SE of three independent experiments. The inset shows *t*_50_ data (time to obtain 50% germinated seeds).

To further verify the effect of GBF1 on *AtCathB3* gene expression in *Arabidopsis thaliana*, the expression profiles of *AtCathB3* and *GBF1* were analysed in two over-expressor GBF1 lines (*oex1-GBF1* and *oex2-GBF1*) and in the homozygous KO line for *GBF1* (*gbf1*), and compared with those in the Wt. The KO *gbf1* homozygous plants as expected, not only presented undetectable levels for the *GBF1* transcripts in germinating seeds but also accumulated *AtCathB3* transcripts at a level of 2- to 3-fold higher than those in the Wt (Fig. 5A, B). In the two *oex-GBF1* lines, the opposite was observed: expression of the *AtCathB3* gene was decreased by >75% compared with that in the Wt germinating seeds. The variation observed in *GBF1* mRNA levels within a given *oex-GBF1* line during seed germination can be explain by the non-homogeneous expression patterns driven by the CaMV 35S promoter during germination, as previously reported (Benfey *et al.*, 1989). However, the GBF1 levels reached were sufficient to negatively influence *AtCathB3* expression. The germination in the *gbf1* mutant seeds was faster than in those of the Wt (*t*_50_=26.1±0.2 versus *t*_*50*_=30.8±0.1), whereas in the two *oex-GBF1* lines, germination was slower and, as a consequence, the corresponding *t*_50_ values were higher (Fig. 5C, inset). These data indicated that GBF1 represses transcription of the *AtCathB3* gene and negatively affects *Arabidopsis thaliana* seed germination.

### *AtCathB3* and *GBF1* are co-expressed in the same cell types in germinating seeds

**Fig. 6.**
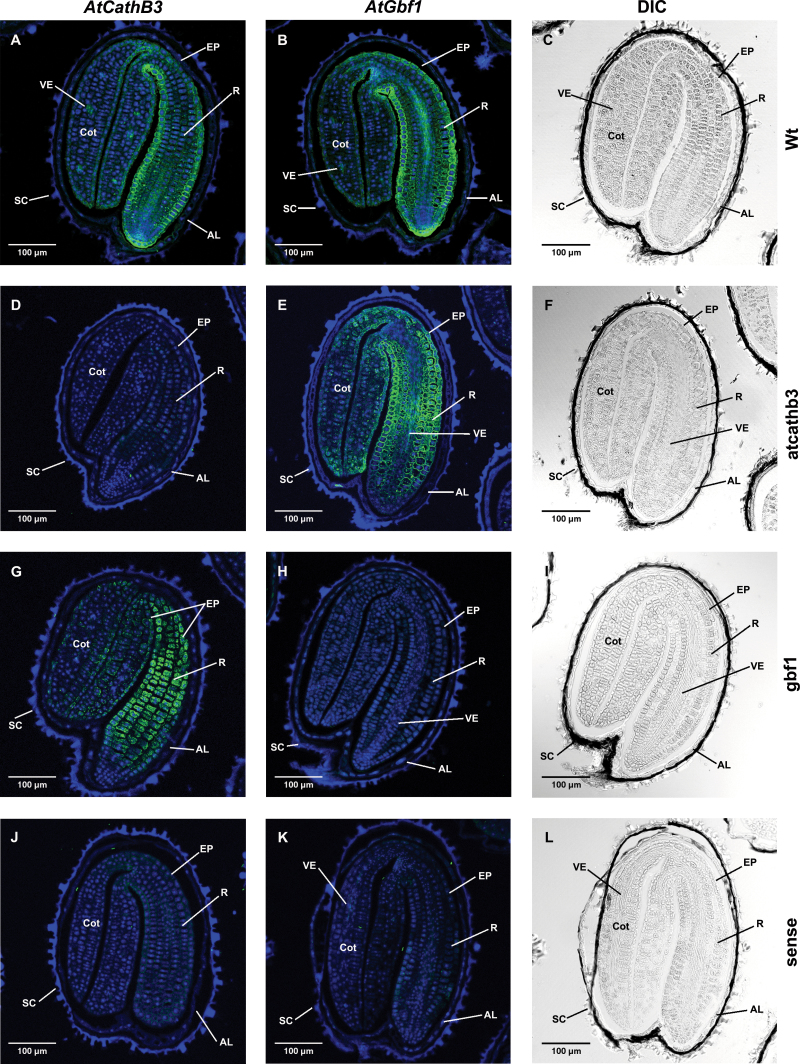
FISH analysis of *AtCathB3* (A, D, G, J) and *GBF1* (B, E, H, K) transcripts in germinating seeds of Wt (A, B) and *atcathb3* mutant (D, E) at 30h and *gbf1* mutant (G, H) at 24h. Results for control sense probes are shown in (J) and (K). All images are merged for the Alexa Fluor 488-conjugated anti-mouse IgG with DAPI staining to reveal the nuclei. (C, F, I, L) Differential interference constrast images. AL, aleurone; Cot, cotyledon; SC, seed coat; R, radicle; EP, epidermis, VE, vascular elements.

If the *GBF1* gene encodes a transcriptional regulator of the *AtCathB3* gene during seed germination, both genes should be expressed in the same cells at the right time. In order to explore this, FISH analyses of both transcripts were carried out in germinating seeds before radicle protrusion at equivalent physiological stages (24h for *gbf1* mutant and 30h for Wt and *atcathb3* mutant; Fig. 6). Longitudinal sections hybridized to specific antisense probes, derived from the 3′ non-coding regions of the *AtCathB3* and *GBF1* genes, showed a strong signal for both transcripts at the epidermis of the embryonic axis and at the incipient vascular elements of the embryo (Fig. 6A, B). As expected, the KO *atcathB3* did not affect the expression of *GBF1* (Fig. 6D, E); however, when the KO *gbf1* line was hybridized with the antisense probe for *AtCathB3* gene, the fluorescence signal increased and appeared not only at the epidermis but also throughout the embryonic axis (Fig. 6G). A similar localization of both transcripts in the same tissue types at same time, as well as the increase in the fluorescence signal for *AtCathB3* in the *gbf1* mutant, is compatible with transcriptional regulation of the *AtCathB3* gene by the GBF1 TF. As expected, no signal was detected in the *atcathb3* and *gbf1* KO mutant seed sections with their corresponding antisense probes (Fig. 6D, H), nor when the Wt seed sections were hybridized with the sense probes as negative controls (Fig. 6J, K). Microscopic analysis of coarse sections (8 µm) of paraffin samples by differential interference contrast (Fig. 6C, F, I, L) showed that the cellular organization of the seed tissues was adequately preserved during sample preparation.

## Discussion

In the genome of *Arabidopsis thaliana*, the CathB-like gene family is represented by three members, *AtCathB1*, *AtCathB2*, and *AtCathB3*. During seed germination, the most highly expressed of these genes is *AtCathB3* (Nakabayashi *et al.*, 2005, and this study). The analyses of the GUS promoter reporter lines, *PAtCathB3::uidA* (–500bp) showed a strong GUS expression in the lower hypocotyl, the hypocotyl–radicle transition zone, and the radicle tip during germination *sensu stricto* (Fig. 2H, I). Similar results were obtained by mRNA *in situ* hybridization experiments of imbibed seeds before radicle protrusion (Fig. 6), where transcripts of *AtCathB3* were localized to the epidermis of the same regions where GUS was detected in the transformed *PAtCathB3::uidA* lines. The lower hypocotyl and the hypocotyl–radicle transition zone have been reported as regions of intense activity of cell expansion upon germination of *Arabidopsis thaliana* seeds. In this region, protein storage vacuoles are rapidly replaced by a central lytic vacuole enabling the elongation of embryonic cells (Sliwinska *et al.*, 2009; Bolte *et al.*, 2011). Interestingly, the protein AtCathB3 has been localized to the vegetative vacuole proteome of *Arabidopsis thaliana* and it is expected to have a similar vacuolar localization in germinating seeds (Carter *et al.*, 2004).

From a physiological point of view, the KO *atcathB3* mutant had a slower germination rate than the Wt, as indicated by their *t*_50_ values (40.4±1.9h vs 30.0±0.1h, respectively; Fig. 3C), indicating that *AtCathB3* positively influences germination *sensu stricto* in *Arabidopsis* seeds. This germination role is different from that in cereal grains where the proteases encoded by *CathB-like* genes are expressed in the aleurone layer and participate in the mobilization of SSPs during post-germination but do not seem to play a role in germination *sensu stricto* (Cejudo *et al.*, 1992; Gubler *et al.*, 1999; Mena *et al.*, 2002; Isabel-Lamoneda *et al.*, 2003).

When analysing the *PAtCathB3::uidA* reporter lines after radicle protrusion (≥36h), GUS expression was found in the cotyledons, enhancing the intensity of the signal with the cotyledon expansion, as well as being detected in the incipient vascular elements of the hypocotyl (Fig. 2). These findings are in agreement with a dynamic model for reserve mobilization that proposes hydrolysis of the bulk of SSPs at the protein storage vacuoles of the cotyledons when those of the embryonic axis are exhausted (Müntz, 2007). The implication of other proteases has been reported, like serine carboxipeptidase III (CPIII) from wheat, in the programmed cell death of the tracheary element differentiation between the radicle axis and the cotyledons to promote vascular development (Dominguez *et al.*, 2002). Our data indicate a role for *AtCathB3* not only in the incipient vasculature development during germination *sensu stricto* but also in SSP mobilization during post-germination.

To gain insight into the *cis*–*trans* transcriptional regulatory code of the *AtCathB3* gene, a phylogenomic approach was used to identify a conserved region of 84bp (the *CathB3-element*) in the promoters of the *CathB3* orthologous genes among several Brassicaceae species (Fig. 4). The power of this approach has been demonstrated in *Saccharomyces* (Cliften *et al.*, 2001), primates (Boffelli *et al.*, 2003), and *Arabidopsis* and related Brassicaceae (Koch *et al.*, 2001; Hong *et al.*, 2003; Adrian *et al.*, 2010; Iglesias-Fernández *et al.*, 2013). Using as a bait the *CathB3-element* for screening a yeast library of approximately 1200 TF ORFs of *Arabidopsis thaliana* (Castrillo *et al.*, 2011), GBF1 (*At4g36730*) was isolated and the specificity of the binding through the G-box element 5′-(A/T)ACGTG-3′ (Schindler *et al.*, 1992*b*) was demonstrated (Fig. 4B, C). GBF1 belongs to the G group of bZIP TFs, which includes five members: GBF1, GBF2, GBF3, AtbZIP16, and AtbZIP68. These TFs share not only their DNA-binding domain but also additional highly conserved motives in the N-terminal region (Jakoby *et al.*, 2002; Supplementary Fig. S3A, B available at *JXB* online). Transient co-expression of the *P35S*::*GBF1* and the *PAtCathB3::uidA* constructs in tobacco leaves revealed that GBF1 acts as a negative regulator of the *AtCathB3* promoter *in planta* (Fig. 4D).

During seed germination (between 0 and 36h), *GBF1* mRNA expression increased up to 2-fold, being the most abundantly expressed bZIP gene within the G group (Fig. 5B, Supplementary Fig. S3C available at *JXB* online). Our results showed that an increment in the *AtCathB3* transcript levels was observed in the germinating *gbf1* mutant seeds, and these mutants displayed a faster germination rate than the Wt seeds. Moreover, the two *oex-GBF1* lines analysed presented not only lower *AtCathB3* expression but also a significantly slower germination rate (Fig. 5). Taken together, these results indicate that *GBF1* is a negative transcriptional regulator of the *AtCathB3* gene during seed germination.

Transcriptional changes occur that are both temporally and spatially separated in the endosperm and embryo of the germinating seeds of *Arabidopsis* (Penfield *et al.*, 2006; Dekkers *et al.*, 2013). *GBF1* is expressed specifically at the epidermis of the embryonic axis upon seed imbibition, and when this gene is knocked out, a delay of radicle protrusion and enhancement of *AtCathB3* expression occurs in the germinating seeds (Figs 5 and 6). Other bZIPs have been reported to be repressors of seed germination, such as ABI5, which provokes inhibition of the endosperm rupture, and its transcripts are localized at the embryonic axis and at the micropylar endosperm in ABA-treated imbibed seeds (Finkelstein and Lynch, 2000; López-Molina *et al.*, 2002; Piskurewicz *et al.*, 2008). Recently, the positive influence on seed germination *sensu stricto* of AtbZIP44 (*At1g75390*; S-group) has been reported, with its mRNAs localized to the endosperm and embryonic axis of germinating *Arabidopsis thaliana* seeds. AtbZIP44 is a positive transcriptional activator of the endo-β-mannanase-encoding gene *AtMAN7* (*At5g66460*). Endo-β-mannanases are important hydrolytic enzymes involved in loosening of the cell walls of mannan-rich endosperm cells during germination of dicotyledonous seeds (Iglesias-Fernández *et al.*, 2011; Rodríguez-Gacio *et al.*, 2012). KO *AtbZIP44* mutants have a statistically significant slower germination rate than the Wt, being affected in two phases of the germination process: rupture of the seed coat and radicle protrusion through the micropylar endosperm (Iglesias-Fernández *et al.*, 2013).

*GBF1* transcripts accumulate during *Arabidopsis* seed germination at the embryonic axis and vascular elements of the embryo at the same time as those of *AtCathB3* (Figs 5 and 6). However, the transcriptional effect of GBF1 upon its target genes can be modified by phosphorylation by casein kinase II, which stimulates its DNA-binding activity (Klimczak *et al.*, 1992; Menkens *et al.*, 1995), and can be also modulated by its interaction with other TFs of the same or different families. It has been reported previously that GBF1 can dimerize with all the members of the G group (GBF1, GBF2, GBF3, bZIP16, and bZIP68) and with HY5 and HYH from the H group, which are master positive regulators of photomorphogenesis, generating positive and negative regulatory responses in light-regulated gene expression and seedling development (Schindler *et al.*, 1992*a*; Singh *et al.*, 2012). During photomorphogenesis, when shortening of the hypocotyls, cotyledon expansion, and development of mature chloroplasts occur, GBF1 plays a regulatory role in cryptochrome-mediated blue-light signalling at early seedling development, acting as a negative regulator of the inhibition of hypocotyl elongation, and as a positive regulator of cotyledon expansion. Moreover, GBF1 may function either as a transcriptional activator or repressor of its target genes, as it negatively regulates the gene encoding ribulose-bisphosphate-carboxylase small subunit (*RBCS*), whereas it is a positive regulator of the chlorophyll *a*/*b*-binding protein-encoding gene (*CAB1* gene, synonymous *LHCB1.3*; Mallappa *et al.*, 2006). This antagonistic role for a given TF in the regulation of different seed genes has been reported not only with bZIPs but also with DOF proteins. In barley seeds, the bZIPs BLZ1 and BLZ2 activate the *Hor2* gene encoding a B-hordein storage protein during seed maturation (Vicente-Carbajosa *et al.*, 1998; Oñate *et al.*, 1999), while they are transcriptional repressors of the α-amylase-encoding *Amy6.4* gene during seed germination (Fuentes, 2008). Similarly, the DOF BPBF (DOF24) activates expression of the *Hor2* gene upon maturation while repressing the *Al21* gene encoding a CathB-like protease induced in the aleurone layers in response to GA during seed germination (Mena *et al.*, 1998; 2002). The interaction between GBF1 and COP1 has also been described, a well-characterized negative regulator of light signalling during photomorphogenesis, and this physical interaction is required for the optimum accumulation of GBF1 in light-grown seedlings (Mallappa *et al.*, 2008). All these data indicate the importance of the combinatorial interaction of GBF1 with other TFs in transcriptional regulation. Whether GBF1 negatively regulates *AtCathB3* expression at the embryonic axis in germinating seeds through its interaction with other bZIPs or other TFs belonging to other families remains to be elucidated.

GBF1 has been also described as being involved in leaf senescence, repressing the expression of genes as *RUBISCO* (*RBCS*) and *CATALASE2* involved in the redox response (Smykowski *et al.*, 2010), and activating a *LHCB2.4* gene encoding a chlorophyll *a*/*b*-binding protein belonging to the chloroplast antennal system (Shaikhali *et al.*, 2012), different from that encoded by *LHCB1.3*, mentioned previously. Interestingly, *AtCathB3* and *GBF1* are expressed at the incipient vascular elements during seed germination and post-germination where these cells undergo a programmed cell death that implicates redox reactions and vacuolization. Our group has previously reported that DOF6 (*At3g45610*) negatively affects seed germination and is also localized at the incipient vascular elements of the embryo (Rueda-Romero *et al.*, 2011). However, DOF6 and GBF1 do not interact in yeast two-hybrid assays (data not shown). The gene encoding the DOF DAG1 is also a negative regulator of seed germination, while its paralogous DAG2 is a transcriptional activator being expressed in the incipient vascular elements of the embryo (Gualberti *et al.*, 2002; Gabriele *et al.*, 2010).

In summary, this work describes a new role for the *AtCathB3* gene, not only during germination *sensu stricto* when the mobilization of proteins occurs at the embryonic axis but also in the post-germination process when SSPs are hydrolysed in the cotyledons, and demonstrates that GBF1 is a bZIP transcriptional regulator negatively affecting *AtCathB3* expression during seed germination. As GBF1 functions in cryptochrome-mediated blue-light signalling during seedling establishment, further investigation is needed to explore the involvement of GBF1 blue-light regulation in protein mobilization during germination.

## Supplementary data

Supplementary data are available at *JXB* online.

Supplementary Fig. S1. Phylogenetic tree with the deduced protein sequences of the *cathepsin B-like* genes of the Brassicaceae species.

Supplementary Fig. S2. Transcription levels of the housekeeping gene (*UBC21*) used in the RT-qPCR analyses, presented as *C*_t_ mean values. Expression of the *ACT8* gene is used for comparison.

Supplementary Fig. S3. *Arabidopsis thaliana* bZIP transcription factors of the G subfamily.

Supplementary Table S1. List of primers used for cloning, for probe synthesis in FISH, and for T-DNA genotyping of mutant insertion lines.

Supplementary Table S2. Major characteristics of the Brassicaceae predicted CathB-like protein sequences.

Supplementary Table S3. Genomic sequences of the *CathB3* gene promoters of the Brassicaceae species used in the phylogenetic shadowing analysis.

Supplementary Table S4. Sequences and characteristic of the primers used in the RT-qPCR analyses.

Supplementary Data

## Abbreviations:

3-AT: 3-amino-1,2,4-triazole
ABA: abscisic acid
bZIP: basic leucine zipper
CaMV: cauliflower mosaic virus
DAPI: 4′,6-diamidino-2-phenylindole
FISH: fluorescence *in situ* hybridization
GA: gibberellin
GFP: green fluorescent protein
GUS: β-glucuronidase
KO: knockout
LUC: luciferase
oex: overexpressor
RT-qPCR: real-time quantitative PCR
SE: standard error
SSP: seed storage protein
TF: transcription factor
Wt: wild type
Y1H: yeast one-hybrid.
