# Supplementary material for: The AtCathB3 gene, encoding a cathepsin B-like protease, is expressed during germination of Arabidopsis thaliana and transcriptionally repressed by the basic leucine zipper protein GBF1
Source: J Exp Bot. 2014 Mar 5;65(8):2009–21. doi: 10.1093/jxb/eru055 (PMC3991739; doi:10.1093/jxb/eru055)
Supplement: Supplementary Data [file supp_65_8_2009__index.html]

The AtCathB3 gene, encoding a cathepsin B-like protease, is expressed during germination of Arabidopsis thaliana and transcriptionally repressed by the basic leucine zipper protein GBF1 — The AtCathB3 gene, encoding a cathepsin B-like protease, is expressed during germination of Arabidopsis thaliana and transcriptionally repressed by the basic leucine zipper protein GBF1 — Supplementary Data 

# The *AtCathB3* gene, encoding a cathepsin B-like protease, is expressed during germination of *Arabidopsis thaliana* and transcriptionally repressed by the basic leucine zipper protein GBF1

## Supplementary Data

Data files

**Files in this Data Supplement:**

- Supplementary Data - Supplementary Data
