# Supplementary material for: The AtCathB3 gene, encoding a cathepsin B-like protease, is expressed during germination of Arabidopsis thaliana and transcriptionally repressed by the basic leucine zipper protein GBF1
Source: J Exp Bot. 2014 Mar 5;65(8):2009–21. doi: 10.1093/jxb/eru055 (PMC3991739; doi:10.1093/jxb/eru055)
Supplement: Supplementary Data [file supp_eru055_eru055_NEW_supplementary_data.pdf]

Figure S1. Phylogenetic tree with the deduced protein sequences of the *Cathepsin B-like* genes of the Brassicaceae species: *Arabidopsis thaliana* (*At*), *Arabidopsis lyrata* (*Al*), *Capsella rubella* (*Cr*), *Arabis alpina* (*Aa*), *Eutrema salsugineum* (*Es*), *Brassica rapa* (*Br*). Bootstrapping values are indicated in the branches.

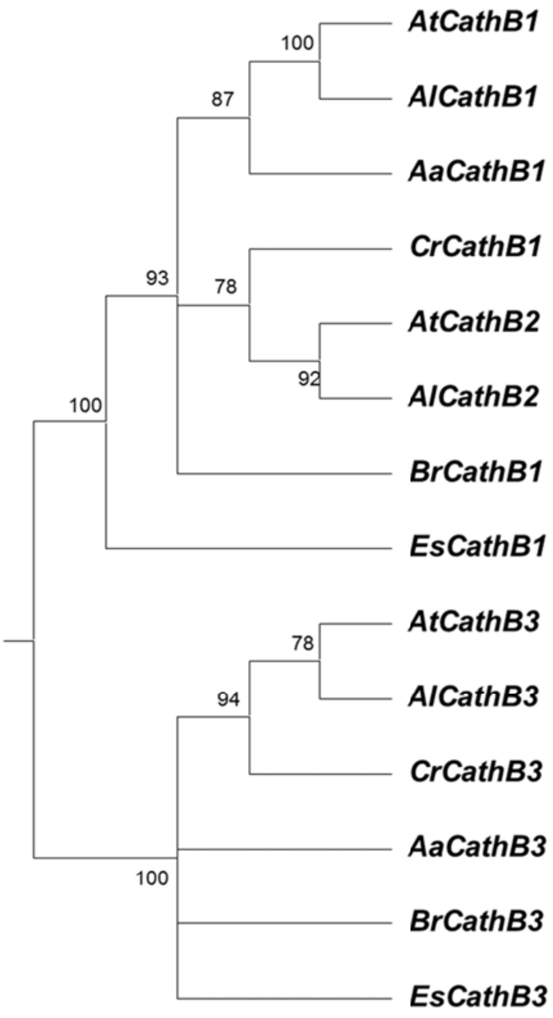

Figure S2. Transcription levels of the housekeeping gene (*UBC21*) used in the RT-qPCR analyses, presented as Ct mean values. The expression of the *ACT8* gene is used for comparison.

(A) Plant material from 5 week old *A.thaliana* plants: roots, rosette leaves, stems and flowers.

(B) Silique development: time intervals 1-3, 4-6, 7-9, 10-12, 13-15 and more than 15 days after pollination (dap).

(C) Seed Germination: 0 (dry seeds), 6, 12, 18, 24, 36 and 48 h.

Data are means ± standard error (SE) of three independent experiments.

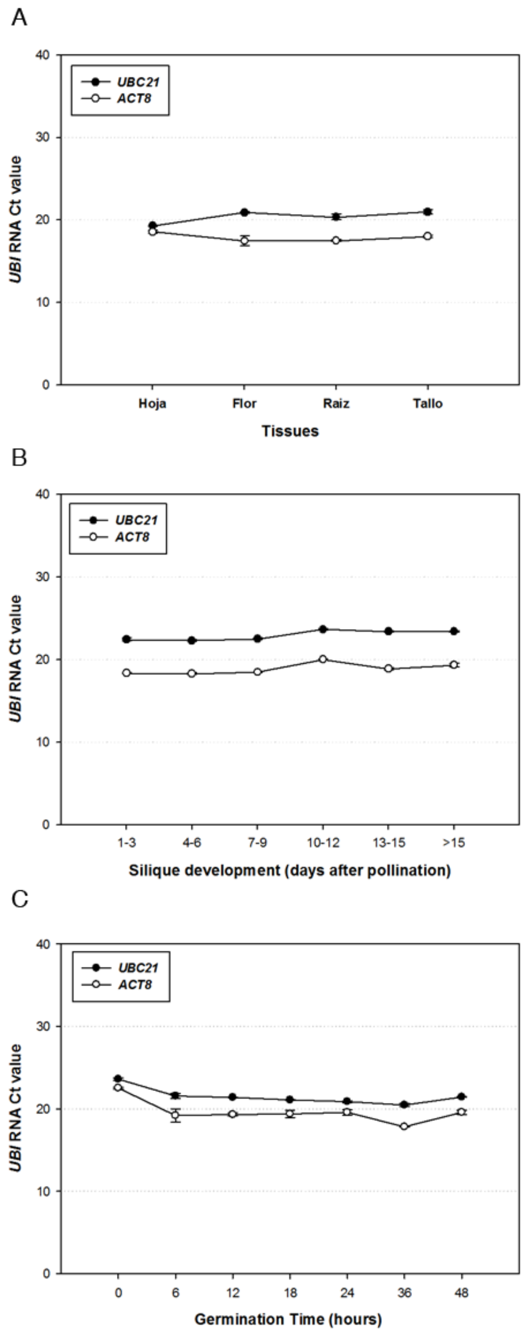

Figure S3. *Arabidopsis thaliana* bZIP transcription factors of the G subfamily.

(A) Phylogenetic tree and schematic distribution of conserved amino acid motives among the deduced protein sequences of the bZIP belonging to the G-subfamily.

(B) Sequences of conserved am ino acids motives (MEME; Bailey *et al*., 2009) of the bZIPs described in (A).

(C) Heat-map representing the expression levels of the G family of bZIP during seed germination (http://www.genevestigator.com/gv/).

A

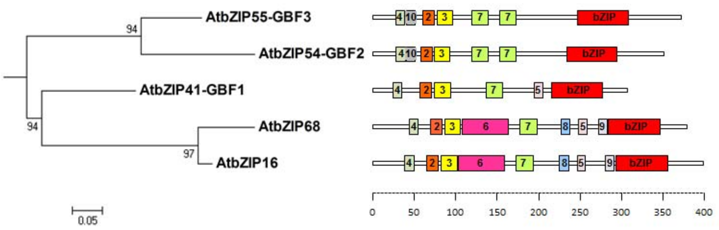

B

Motif	E-value	Consensus sequences
1	5.2E-097	[DN][ED][RK]E[LIV]KR[QE][RK]RKQSNRESARRSRLRKQAE[CT][ED][EQ]L[AQS][QRV][RK][VA][ED][AVS]L[NST V][AEGN]EN[MQST][SAN]LR[ASD][EK][LI][NGQ][KQR]L[KNS][SEN][EQK][CSY][ED][KE]L[RKLT][AGLST][EA]N
2	1.1E-021	P[HP]PYMW[GAN][VAPS][QP][HS][MHP]M[MPS][PA][YP][GY]
3	3.5E-017	[PY][YA][PVA][APTV][MFY][YCP][PH][PG]G[GA][VM]YAHP[SG][MILV][PQ][MP]
4	3.8E-012	[VYA][THPY][PDHQ][DW][WQ][ASN][AGS][MF]QAY[YS]
5	1.8E-009	TNLNIGMD[YL]W
6	2.4E-006	YPYSPYAMPSPNGM[AT]E[AV]SGNT[GT][GS][GV][IT][DE]GD[AG]K[PQ]S[DE][GV]KEKLPIKRS[KR]GSLGSLNMI[IT]GKNNE[AP]GKN
7	4.3E-004	[DI][DG][SAT][PS][ADET][GKN]S[DAT][GA]N[ST][DQ][NH][DG][SFL][GM][KS][KGR][LH][KDN][EG][FK]D[GA] [EL][AT][AM]S[EIL]
8	1.1E+001	[IN]LPV[NS]QTV[AP]IM
9	1.1E+002	DG[GS][HQ]SQPWLQ
10	1.6E+002	PRV[AG][IM]P[PQ]YYN

*Motif 1 spans the putative bZIP domain

C

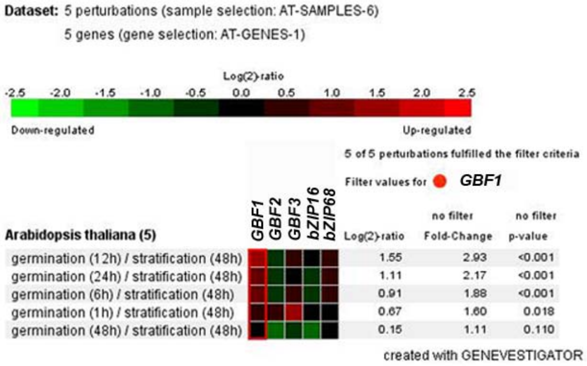

Table S1. List of primers used for cloning, for probe synthesis in FISH and for T-DNA genotyping of mutant insertion lines.

PRIMER NAME	LOCUS	SEQUENCES 5’ -3’
**Cloning primers**		
S-PAtCathB3	*At4g01610*	AAAAAAGCAGGCTTCCGAAGACTATTTTGATC
AS-PAtCathB3	*At4g01610*	CAAGAAAGCTGGGTCAGCCAGAAAACAGA
S-PAtCathB.SE-Y1H	*At4g01610*	AGCCCGGGTAATGACGCAACGTG
AS-PAtCathB.SE-Y1H	*At4g01610*	AGTCTAGAAAAGAAGATCTTAGCC
AS-PAtCAthB-SEm-Y1H	*At4g01610*	AGCCCGGGTAATGACGCAACTGTGCAATCATGTATA
attB1	*GATEWAY*	GGGGACAAGTTTGTACAAAAAAGCAGGCTTC
attB2	*GATEWAY*	GGGGACCACTTTGTACAAGAAAGCTGGGTC
**Fluorescence *in situ* hybridization (FISH) primers**		
S-AtCathB3-IH	*At4g01610*	TCTAGAGGTTCGAATGATCTTCCGGT
AS-AtCathB3-IH	*At4g01610*	GGATCCCGGGTGATCAAAAGACGAAT
S-GBF1-IH	*At4g36730*	AGAAGCTTAATGTGATAAGCTCAAGTCT
AS-GBF1-IH	*At4g36730*	CTCCATCCAGCTTCTACAGTCTTGTAGG
**Genotyping primers**		
LP-AtCathB3	*At4g01610*	TTGTGTGTGTGTGTTGACTGC
RP-AtCathB3	*At4g01610*	AAAACTTACATCACCCCAGCC
LP-GBF1	*At4g36730*	TCAGAATCCGATTCCAATCAC
RP-GBF1	*At4g36730*	ATGAGAATGCCAATCAACAGG
LBb1.3	*LB of the TDNA in SALK lines*	ATTTTGCCGATTTCGGAAC

Table S2. Major characteristics of Brassicaceae predicted Cathepsin B-like proteins*.

Protein name	Locus	Protein size (aa)	pI	Mw(Da)	Peptidase C1A Propeptide[1]	Peptidase C1A papain C-terminal[1]	Cysteine active site[1]
**AaCathB1**	*Contig13570*	350	6.54	38901.23	45-87	105-328	127-138
**AaCathB3**	*Contig16048*	357	6.09	39435.92	45-87	105-339	127-138
**AlCathB1**	*918880*	359	6.27	39629.19	43-85	103-337	125-136
**AlCathB2**	*918881*	360	6.66	39892.41	44-86	104-338	126-137
**AlCathB3**	*490415*	359	6.27	39409.72	45-85	103-337	125-136
**AtCathB1**	*At1g02300*	379	6.80	42260.18	43-85	103-358	NA
**AtCathB2**	*At1g02305*	362	6.48	40030.51	46-88	106-340	128-139
**AtCathB3**	*At4g01610*	359	5.79	39414.68	43-85	103-337	125-136
**BrCathB1**	*Bra030498*	357	6.55	39501.72	41-83	101-335	123-134
**BrCathB3**	*Bra036321*	358	5.65	39060.43	42-84	102-335	124-135
**CrCathB1**	*Carubv10009537m*	360	7.26	39693.32	44-86	104-338	126-137
**CrCathB3**	*Carubv10001253m*	359	6.09	39299.70	43-85	103-337	NA
**EsCathB1**	*Thhalv10008009m*	360	7.20	39938.23	44-86	104-338	126-137
**EsCathB3**	*Thhalv10028753m*	362	5.66	39793.07	46-88	106-340	128-139

*From the Phytozome database, three non-redundant Cath B-like deduced protein sequences from *Arabidopsis thaliana* (*At*) and *Arabidopsis lyrata* (*Al*), and two from *Brassica rapa* (*Br*), *Capsella rubella* (*Cr*), *Eutrema salsugineum* (*Eh*) have been compiled. Two putative orthologous genes present in the *Arabis alpina* (*Aa*) genome has been also included in these analysis. aa: amino acid number; pI: isoelectric point; Mw: Molecular weight; [(1)]: amino acid position.

Table S3. Genomic Sequences of the CathB3 gene promoters of the Brassicaceae species: Arabidopsis thaliana (At), Arabidopsis lyrata (Al), Capsella rubella (Cr), Arabis alpina (Aa), Eutrema salsugineum (Es), Brassica rapa (Br), used in the phylogenetic shadowing analysis. The sequences shown span from -1000 bp upstream from the ATG codon to the end of the first exon of the CathB3 gene (red bold). Underlined letters: promoter sequences for CathB3 genes; red letters: translated regions; blue letters: untranslated regions; black letters: non-coding sequences

>*AtCathB3* Chr4:693,695..694.934

GAGAGTGTTTCAACGAGAGTTTGGTGTTTTAGCTGTAGAGAAACTTTTAGATTC
GTTTGTTTGCTACATATCGACTACTTCGGGACCAGTGACCGGAGTGATATACAT
TTCCAACAGAAGAATCGCTTTTTGTAGCGACTATGCCATTCGTCTTCCTTCTTCT
GCCGGTGGAAACGGTGTTGCGGCATATTACAAGGTTAGACCATGAGTTTCAAA
CAATATTTTTTGTAATAGAAGTTTGGTTCATACTTAAAAAAAAAGATCTAAGTT
GGTATTGGTAACAAGAATTGAATTGTTCTTTGTGAAATAGCTGTTTTCATTTTGT
AAAATAAGAAAATAATAATTTGGTTTCTAAAGATTAGTGACTATATAATATATT
GAGCAATAATAAAATTTATATGTGATATTGTATTATAATGTTGTATATTGATAG
GTGGTGATGGAGTGGGAGAAGATAAGTAGCATTAGTTCATCAACGAACGTGTT
GAAGCCAAGTGAGAGATACGTGCATATGGTGACACGAGATGGGTTTGAGTTTT
GGTTCATGGGCTTTGTCTCCTACATCGATGCTTTCAATTGTCTCAACAAAGCTCT
CCTCAATTCCCGTTGTAATCATGCCATGTAATCAAATTCTAATTTTCTTATCAAA
ACTACGAAGACTATTTTGATCAGATCTCATAAAATATCAAAAAAAAAAATTAC
AAGTTTACCACCAAAATTATTTATCTGATTCATAACTTTGACCAACCAAAAAAA
TACAAAATTGGGATAACTGAGTTACACAGGTAACTTATTGATGCTTGTTAAATT
GTCATTGCGATTTCGTAACAAAGGGTCCAACTCCAATCCCATAAAAAATTACG
AACGATGTGTAATGACTAATGACGCAACGTGGCAATCATGTATACCTTCAACG
GCCTAGAATTCTGTAGATTGCATCAATCAACGGCTAAGATCTTCTTTTGGCTCT
TAACAAAGTCATTGGCTGATTCAGTTTCCCAAGAAATGTCTCTGTCATTGTGCA
GTAGATAAGCCACTCTCTCTCTCTTTCTCTCTTTTTGCTATTATAAAGAAAAAGA
TTCATTCTCCCACCATTGAGGTTTAACTTATACAAACAGATCTTTTTGAAATTGG
ATTCATCTAAGAAGCAGACTCTGTTTTC**ATG**GCTGTTTACAATACCAAACTCTG
TTTGGCCTCTGTTTTCTTGCTCTTAGGGCTTCTCTTGGCTTTTGACTTGAAG

>*AlCathB3* scaffold_6:24,227,748..24,228,962

GCTCAATTTTTGATCTAATTATATTACTCATTCGATTTGACTTGACCCGATCTAA
TTCTATTACCCATTCGATATGGACTTGTCCTCGATCTAATTCTATTACCTAGTCG
ATCTGAACTTGTCCTCGATCTAATTCTATTCCAGTTGTCCCCGAGTAAATCCAG
ATCTCTGGCGGGCAAAACTACACCATCTGGCGATGGACAGTCAAGAGAATTGG
ATGATAAAAATCATCACGCTTAATTTTTGATCTAATTATATTACTCATTCGATTT
GACTTGTCACCGATCTAATTCTATTACTCATTCTATCTGAACTTGTCTCCGATCT

AATTCTATTACTCATTCGATATGAATTTGTCCCCGATCTCATTCTATTACTCATT
CGATCCGAACATGTCCCCTCTTTTATCTGATATATTAAAAGAGCAATTAATGGT
CTAACTCTACTCTATTATCATTTTTATTAATTAAATTCAATTATCTAGCAAATGT
AAATGTGCAGCATTTATTGCAGACCTACCTGCTCCTTCTTTGGAAGCGCCGGAC
GAAACTGTCTCTGTCTCTGACACAGACTCTGTCCCAATCAGGGGTATTTTGGTC
CACAATTCCGCATGTGCTATTGAAATGTGGTAGATTTTGCAATATTCACATTCA
TAATTTGGTCTTATAGTAAATCAACATTAACATTCGTTTTTTTTTTTCCGACTAA
ATCAACATTAACCTATAACCCAAAGAATAAAAAAAAATTTGGGTAACTAAGAG
TTACAAACTTACACAAGTAACTTATCTGATCGATGGGTGTTAACTTGCCATTGA
GATTTCGTAACAAAGGGTCCAACTCCAATCCCATAAGATACTATTACGAAAGA
TGTGTAATGAGTAATGACGCAACGTGGCAATCATGTATACCTTTAACGGCCTAG
AATTCTGTAGATTGCACCAATCAACGGCTAAGATCTTCTTTTGGCTCTTAAACA
AGTCATTGGCTGATTCAGTTTCCCAAGAAATGTCTCTGACATTCTGCAGTAGAT
AAGCCACTCTCTCTCTCTCTGAAGATTCATTCCCCCACCACTGAGGTTTAACTTA
TACAAATAGATCTTTTGGTTTTTGATTCATCGAAGAAGCAAACTCTGTTTTC**AT**
GGCTGCTTACAATACCAAACTCTGTTTGGCCTCTGTTTTCTTGCTCTTAGGGCTT
CTCTTGGCTTTTGACTTGAAG

>*CrCathB3* scaffold_6:15,984,537..15,985,614

ACAATATCATTTACAAAGCATCTGAGAAAATGTCAAGAAGGGTTTCTCAATGG
TATTTTTTGAACTGTCTAAAACTTATGGTGTTTTGGAACATCAAAAACTATATCT
TTGGCATCTTAGCCTTTAGGTATTGTTTCTAAATGCTACAAATGTTATGCTGGT
GCTGCTGATATAGGTGGTGATGGAGTTGGAGAAGATAAGTAGCATTGTTTCAT
CATCGAACGTGTTAAAGCCGAGTGAGAGGTATGTGCATATGGTGACACGAGAT
GGGTTTGAGTTTTGGTTCATGGGCTTTGTCTCCTACACCAATGCTTTCAATTGTC
TCGACAAAGCTCACCTCAATTCCCACCACTAATGATGTCTTGTAATCACATTCT
AGTTGAGAATTTTATTATCACTAATAATAATTCTTTCTTGTCTAGTCATATAATA
AGTATGTAACTTGTAAGAACTCTGTTGCCATGGTCTGGAATAAAACTTTGTGAG
TATAAAAGTGTAAAAACCCTACTCGTTAGATAAATTAGAAGTTTAGCTAGGAT
GTATTGGTAAATCAAGAACGAAATTAAATTTGACTTCTAGGATAAATTGAGCT
ACAAAGTATGTAAACTTTAATGATTAGTGGTTGTTATTTGTTAACTTGCCGAGT
TGGTATAGCGATTTCGTAACAAAGGGTCCAACTCCAATCCCATAAGATATTACG
AACGATGTGTAGTGAGTAATGACGCAACGTGGCGATCATGTATACCTCTAACG
GCCTAAAATTCTGTAGATTGCACCAATCAACGGCTAAGATCTTCTTTTGGCTCA
TTGGAAATAATAGTCATTGGCTGATTCAGTTTCCCAAGAAATGTCTGTGTCATT
GTGCAGTAGATAAGCCACTCTCTCTCTCTCTCTTTTTGGTATTATAAAGTAAAG
GAGATTCCCCCACCACTGAGGTTTTTACTTGTCCCAAAGAGATCTTTTGGTTTTG
GATTCCTTGAAGAGCAAAACTCTGTTTTC**ATG**GCTGTTTACAATACCAAACTCT
GTTTGGCCTCTGTTTTCTTGCTCTTAGGGCTCGTCTTGGCTTTTGACTTGCAG

>*AaCathB3* contig-16048

TAAAAATTTGTTTTGACCAAAAAAAAAAGTCTATAAAAAATTGTTTTTCTAAAT
AATTTGTTTTGTTTGCTCTATAAATTATTGCATTTTAAACTAACAACGACATGGC
GTGGTCGCCTTTATATAAATGGATCCACATATATGGAAAAAAACGACGCGAAA
AAAGAAGACGACATTTTGGTTGAGCTCATCCCCAAATGAAATTTGGTATATGA
GATCAAAATCATATCGTCTGTGAAAATATTTGGAAAGAAGGATTTAAACTGCT
ATCTTTTTAATTTAATTTTTTTGTAATCAAAATTTCAGTTTAGTTATAATCGCAT
ATCTCTACAAATTCAAATTTGTTTTTCAGGTTGATTGTTTTAAATCTTAAACATT
ATTATCTATTTTTCTCATGTGGGTGAAAAATGAAATTTCACTTTTTAACAACAA
GAACAAAAAATTGAAACAACTATAATTTACTTGGGCTATTATCCCAATTTATTA
CTTTATCCCATAGAATCATCCATATTTTTTGCATAATCCAATACATAACACAAT
ATAATATTGCACCTTTATTAGTGGTTATGGGTCTAATGGCGATTTCGTAAGAAA
TGATCCAACTCAATCACAATACGTTAATACGACGACGTGTAATAACACAACGT
GTCAATCATCAATAGCTTGAACGGCCTAGATTTCTGTAGCTAAAACCAATCAAC
GGCTAAGATCTCTTTGCTCTTAAAAAAACTAAATTATCATTGGCTTATTCACTTT
CCCAAGAATTGTCTTTGTCATGCTTCACAAGATAAACCCAATCTCTCTTTCATTA
TATAAAGGAGATTCCCATTTTAGTTGGCTTTACTTTCTACACACCAGGATCTTG
ATTTTTCTTGAGCTTGGAGAGATTC**ATG**GCTGATTCTATAACCAAACTCTGTTT
AGTCTCTGTTTTCTTGCTCTTTGGACTTGTCTTGCAGGTAAAGCTTTCT

>*EsCathB3* scaffold_3:693,808..695,035

TTATAATGTATTTAAAGGTGGTAATGGAGTTGGAAAAGGTAAGGAGCATAAGC
TCATCGTCGAACGTGTTAAAGCCAAGTGAGAGGTATGTGCATGTGGTGACGCA
AGATGGTTTTGAGTTTTGGTTTATGGGATTCGTCTCCTACATCGATGCTTTCAAT
TGTCTCAACAAAGCTCGTCTTAACTCTCATTGTGTTTCTGGCATGTAAACCAAA
TTCTAAGACTTTTCTTATCACTAATAAGTCTTTGTTGTCTACTCATATGTACAAC
TTGTATTGTAATCATTTGGGTTCGCCATGGTGTAGTAACAAAAGAATTGATTTA
CACAAAAGTAACTTGTTTACGGTCGTTATTAGAGATGAGTGCGTTATTTGATAG
AGTGAATTCTATTATCTGTTGCTATCTATTATATTACTCTCTATAATTTTAGTGG
TAAACGTGATCCACTAGTTTCGTGTGGATCAGCACTTGTAAGAATATATGATTT
GTTTGGTTAACTTAGTAAAATAATTAAAAGTACTAGAAAAAAAAAAACTCACA
AAGAATTTGCAATATTCGTAGAGAGCTTTTTTTCTTCTTCTTAATTTACACCTTT
TAACATACTTTCTTATTTTGATAATAAAGAGTCTGATGAATACAACAAATTCAG
GAAATTAGTATCTAAATCTAATAATTAAAAATTTATATAGATACATGTATAAGA
GTGAATCATCATATATTAGTTGCTTGCTCCATAAAATTACAATTATCTGAAATT
TCCGCATAAATTCAAAATAGATAACAAACTGATATTTTACCTTTAGTGGTTATG
GCGATTTCGTAATGAAGGGTCTAACTCTAATCGGACGACGTGTAATGGTTCAAC
GTGGCGATCATGTATAGCTTGAACCGCCTAGTTTTTCGTAGATGAGAACCAATC
AACGGCTAAGATCTCATTTCCCCAACTCTCTTGGCTCTTTAAAAAAGTCATTGG
CTCATTCAGTTTCCCAAGAAGTGTCTCTGTCATTGTGCAGTAGATAAGCCACTC
TCTCTCTATATTATAAACGAGATTCTCCCATTAGTGAGGTTACTTCAACAAAGA

GATCTTTTGATTTTCTGGGATTCATCGAGAAGAGTTTGAAAGTGAAAAAGAGA
GATTCCC**ATG**GCTGATTCTACCACCACCACCAAACTCTGTTTGGTCTCTGTTTTC
TTACTCTTAGGGCTTGTCTCCTCGTCTTTCGACTTGCAG

>*BrCathB3* A09:560,816..561,890

CGAGATGTAAAAAAATAGTTTGGGCCGCAATATGTTATAAGCAGTTAGAGAAA
ATCTTATTGGATGAGTAAAGTCCAGTGTATGTTGATCGAAACAAACATCAAGTC
TAAAGTAAAATGAAGCATATAATACTGAGCAACCAAAACACGTGTTGTATACT
TAAATTTTAATATGTGCAGAGACTTTTATAATTATTATTCGTTAACCTTAATTTT
TCATACTTCCTCAGGGCTTGAGTTTACTTTCTGGCCACATGAATTTCTGGAAGA
CTAACCCATAAATTTAATTGGGCTCGGATATATGTTGCAATAGTTTATTTTTGG
GCTGGAGAATATGCTAGATGACATGTCATATTGATTGGTCCTGAATATTTTAGG
TGAAGTGGACACTCTTAGAAGCCACAAAATTAGTTACTTTTATATAGTAGGATG
ATGAATACAAATTACGAAAATATGCATACAAATTTTTTCTTTTTGTCGAAAATT
TGCATCAGTTTAAGATGATGATTTTTTTTTTTGTCAAAAATGATGAATACAAAT
TATTATTTTCAATACTAGTTGTATTATATTTACTTGCTCCGTAAAATTACAAAAT
AACCAGAATTTTCCGCATTAAAAATCAACAAAAATGGAACCAATATTAATCTA
CCACTAGTGGTTTATGCGGATTTCGTGACGAAGAGTCCAACATCCAAATACGA
CGACGTGTAAAACTCTAAATGACGCAACGTGTCCATCATGTATACCTCGAGAA
CGCCTAGTTTTCCGTAGATGAGAACCAATCAACGGCTAAGATCTCAATTCACTT
TCCCAAGAAAAATCTCTGTCATTTCTTCAGTAGATAAGCCACTCTCTCTCTCTCT
CTCTCTCTCTCTCTATCTCAATTATAAAGAAAAAAAGATTCTCCCATTACTGAG
GTTGCGTCCATTACAAGATCTTTTAGAGTTCTCTGAGATTCATCGACTTGAGAA
AGTGTGAAAAGGAGAAGAGATTCCAAAACC**ATG**GCTGCTGATTCTACAACCAA
ACTCTGTTTGGTCTCTGTTGTCTTGCTCTTAGGCCTTGTCTCCTCCTTGCAG

Table S4. Characteristics and sequences of the primers used in the RTqPCR analysis.

RTpPCR primers					
PRIMER NAME	SEQUENCES 5' -3'	LOCUS	PRIMER EFFICIENCY(%)	AMPLICON DISSOCIATION Tª
S-CathB1RT<br>*AS-CathB1-RT*	AGTACCACGGTGTAGTAACC<br>GATTGATTCTGTATGCGCCG	*At1g02300*	90.05%	74.9ºC
S-CathB2-RT<br>*AS-CathB2-RT*	CACCACGGTGTAGTCACTG<br>GACCTTGTACGCACTA	*At1g02305*	92.63%	75.7ºC
S-CathB3-RT<br>*AS-CathB3-RT*	CCGGTTGCATCGGTTTAAG<br>GTCACCGGAATCTTTTACTGG	*At4g01610*	93.15%	71.8ºC
AS-GBF1-RT<br>*S-GBF1-RT*	GCAGGCCGAATGCGAACAAC<br>AGCTACAGCCTCTGCTCCAA	*At4g36730*	99.30%	75.1ºC
S-UBC21-RT<br>*AS-UBC21-RT*	GCTCTTATCAAAGGACCTTCGG<br>CGAACTTGAGGAGGTTGCAAAG	*At5g25760*	95.80%	76.3ºC
S-ACT8-RT<br>*AS-ACT8-RT*	GGTCGTACAACCGGTATTGT<br>GAAGAGCATACCCCTCGTA	*At1g49240*	Graeber *et al*., 2011	
